# Latest evidence of microwave ablation for papillary thyroid microcarcinoma compared with surgery: A systematic review and meta-analysis

**DOI:** 10.3389/fonc.2023.1088265

**Published:** 2023-02-07

**Authors:** Jie Feng, Yizhou Jiang, Yiyan Feng

**Affiliations:** ^1^ Department of Ultrasound, Xiamen Haicang hospital, Xiamen, China; ^2^ Department of Thyroid and Breast Surgery, Xiamen Haicang hospital, Xiamen, China

**Keywords:** microwave ablation, papillary, thyroid, microcarcinoma, PTC

## Abstract

**Background:**

The most typical thyroid gland malignant lesion is papillary thyroid cancer (PTC). In many nations, the prevalence of thyroid cancer (TC) is rising, particularly papillary thyroid microcarcinoma (PTMC). Microwave ablation (MWA) has been gradually carried out in some patients with benign thyroid nodules, some low-risk PTMC, and metastatic lymph nodes in the neck. The role and safety of MWA remain controversial topics. So we conducted this study to provide the latest evidence of MWA for PTMC compared with surgery.

**Methods:**

Patients’ postoperative outcomes (duration of hospital stay and hospitalization expenditures), intraoperative outcomes (surgery time, blood loss, and incision size), and follow-up outcomes were all examined (complication rate, recurrence rate, and lymph node metastasis). The effectiveness and safety of MWA versus surgery for PTMC patients were compared using the weighted mean difference (WMD) and odds ratio (OR).

**Results:**

In total, we included 7 articles (7 trial comparisons) which contained 1, 567 PTMC patients. The results showed that MWA had significant advantages in operative time (WMD = -53.47, 95% CI: -67.62 to -39.32), postoperative hospital stay (WMD =-4.59, 95% CI: -6.40 to -2.77), hospitalization costs (WMD= -70.06, 95% CI: -90.93 to -49.19), blood loss (WMD =-28.07, 95% CI: -33.77 to -22.38), incisions size (WMD =-59.69, 95% CI: -67.79 to -51.59), and complication rates (OR = 0.28; 95% CI: 0.18 to 0.42) compared with surgery. It also showed that recurrence rates and risk of lymph node metastasis are similar to surgery.

**Conclusions:**

For PTMC patients, MWA could be an efficient, safe, and affordable therapy.

## Background

Clinically, thyroid nodules are typical. About 7–15% of thyroid nodules are cancerous, while the majority are benign (1). The most typical thyroid gland malignant lesion is papillary thyroid cancer (PTC) (2). The development of papillary thyroid microcarcinoma (PTMC) within the thyroid gland is responsible for around 50% of the rise in the incidence of thyroid cancer that has been observed in numerous nations. The associated mortality rate, however, was either unchanged or lowered (3, 4). PTMC refers to papillary thyroid carcinoma with a maximum diameter of ≤10 mm (5). The characteristics of low-risk PTMC are as follows: (I) the primary tumor is a single lesion (II) the maximum diameter of the primary tumor is less than 10mm. (III) Rather than being close to the thyroid capsule or trachea, the primary tumor is located in the core of the thyroid gland. (IV) there is no regional lymph node metastasis after evaluation (6).

Guidelines from professional societies recommend surgery as the ultimate treatment (7). Although thyroid lobectomy is effective, various complications (such as hypoparathyroidism and damage to the laryngeal recurrent nerve) can happen following surgery, which can significantly impact the quality of the patient’s life (8). Microwave ablation (MWA) has shown good efficacy in the treatment of PTMC, especially in low-risk PTMC (9). Guided by imaging technology, MWA uses a microwave magnetic field to coagulate, and dehydrate eradicate tumor tissues to achieve the purpose of treatment. In recent years, MWA has been gradually carried out in some patients with benign thyroid nodules, some low-risk PTMC, and neck metastatic lymph nodes. MWA is associated with less blood loss, shorter procedure time, and shorter hospital stay compared to open procedures. In order to evaluate the efficacy and safety of thermal ablation and surgery for the treatment of low-risk PTMCs, Kim et al. conducted a meta-analysis. Both thermal ablation and surgery are effective and risk-free options for the treatment of low-risk PTMCs, with thermal ablation having a lower complication rate. Only four studies, totaling 653 individuals who received thermal ablation or surgery, were considered (10). Thermal ablation for the treatment of PTMC was compared to routine surgery in a comprehensive review and meta-analysis for cost, efficacy, and safety. They found that, for the treatment of PTMC, thermal ablation offers a typically secure and affordable alternative to surgery (11). Microwave, radiofrequency, and laser ablation are all types of thermal ablation, and so on. Our study mainly focused on the effect of MWA in PTMC. There was no evidence of differences in disease progression and major complications between MWA and surgery (12). The role of MWA remains a controversial topic (13), and many related studies have been published recently So an updated meta-analysis is necessary.

## Methods

### Literature search strategy

The Cochrane Library, Embase, PubMed, and Google Scholar were all completely searched as of October 2022. The following search terms were employed: Surgery, microwave ablation (MWA), and papillary thyroid microcarcinoma are the first three (PTMC). The Boolean operators “AND” and “OR” were used in the technique to combine all of these terms. The language of the literature search was limited to English. Two of the authors designed the search technique, which also included a literature review and reference list screening, but it should be highlighted that not all pertinent research may have been included.

### Study selection

The studies incorporated into our meta-analysis met the following requirements: (I) Patients from PTMC who are at the stable stage are also included, as is a MWA test group., (II) included a surgery control group, (III) observed the primary outcome indicators of intraoperative outcomes (such as length of the procedure, blood loss, and incision size), postoperative outcomes (like the length of the hospital stay and the cost of the hospital stay), and follow-up results (such as the frequency of complications, recurrences, and lymph node metastasis), and (IV) used either a prospective or retrospective study design. Our meta-analysis did not include the following studies: (I) there wasn’t enough data, (II) the research compared other thermal ablation, such as RFA and laser ablation, (III) there weren’t enough therapies to assess, and (IV) The research was either an abstract, review, or duplicate publication.

### Data extraction and quality assessment

The data were separately extracted by two researchers, and any disagreements were resolved through discussion with a third reviewer or by reaching a consensus. The planned data components from each investigation were extracted using a structured data abstraction form. The information that was gathered included information on the study’s authors, design, patient characteristics (such as age and gender), duration of the research year, and follow-up period. Since all of the included publications were randomized clinical trials, the risk-of-bias evaluation was finished using the Cochrane Risk of Bias 2.0.

### Sensitivity studies

In accordance with the characteristics of the included research, sensitivity analysis was used to investigate the origins of heterogeneity. After properly removing one of the included studies, the pooled analysis was then repeated. The new pooled effect size was contrasted with the initial pooled effect size to ascertain how the excluded studies affected the pooled effect size.

### Statistical analysis

Stata (version 17.0) was used in our study’s meta-analysis to calculate the various impacts on patients with PTMC in the MWA and surgery groups. In essence, we used the odds ratio (OR) to account for the pooling effects of binary variables and the weighted mean difference (WMD) for continuous variables. The I^2^ statistic, which measures discrepancies in the research findings and indicates the percentage of the total variance in study projections that may be attributed to heterogeneity rather than sampling mistakes, was used to evaluate the disparity in the statistics between the studies.If there was no statistical heterogeneity, the meta-analysis was conducted using a fixed-effect model. (I^2^ ≤ 50) between the included researches. If there existed statistical heterogeneity, however, a random-effect model was employed. (I^2^ > 50). If there were more than 10 included studies, the Egger’s test and a funnel plot were used to investigate potential publication bias 10.

## Results

### Search process

**Figure 1:**
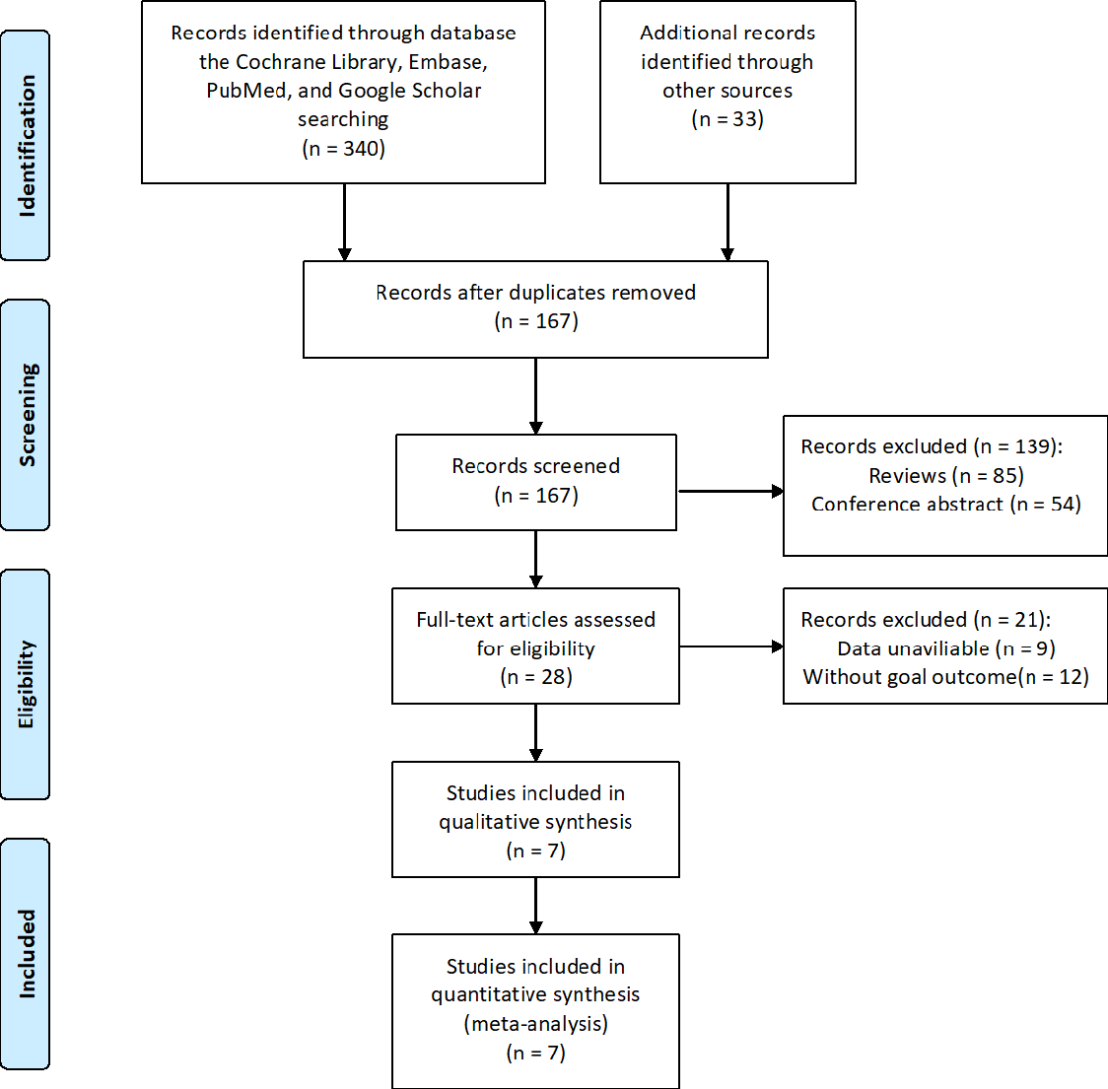
Flow diagram for eligible studies.

There were 373 distinct studies found in the literature search, and after duplicate files were removed, 167 papers were checked for eligibility. Further 139 were eliminated from consideration after the titles and abstracts were checked. To identify the kind of paper, the relevant outcome data, and the research design, the entire texts of 28 articles were examined. Ultimately, data was extracted from 7 studies (14–20) that were included in our meta-analysis. **Figure 1** displayed the method for conducting a literature search, the rules for inclusion and exclusion, and the final studies that were included.

### Characteristics of included studies

**Table 1** provided a comprehensive summary of the meta-analysis papers. The 7 studies were all randomized prospective or retrospective studies. The meta-analysis comprised 1,567 patients in total (779 MWA patients and 788 surgery patients). The studies’ sample sizes varied from 87 to 644. The majority of the research publications on MWA for PTMC have been published after 2018, as it is a relatively new therapy.

**Table 1:** Main characteristics of the included studies in the meta-analysis.

Study	Study design	Publication year	No. of		Gender (M/F)		Age (years)		Mean diameter of nodule		Follow-up (months)
			patients						(mm)		
			MWA	OS	MWA	Surgery	MWA	Surgery	MWA	Surgery	
Li et al	R	2018	46	46	16/32	13/33	43.63±9.27	49.59±9.0	4.49±1.55	4.29±1.37	42
Xu et al	R	2018	41	46	12/29	16/30	45.8±10.2	46.2±11.5	8.87±1.01	8.13±1.22	–
Chen et al	R	2018	49	40	9/40	5/35	44.88±11.04	45.78±13.74	–	–	24
Li et al	R	2019	168	143	36/132	29/114	47.36±10.75	49.18±11.41	–	–	12
Zu et al	R	2021	320	324	83/237	77/247	44.99±10.62	43.91±11.47	4.99±1.93	5.11±1.88	84
Wang et al	R	2021	63	83	12/51	24/59	43.56±14.17	43.32±10.88	4.5±1.1	4.5±1.1	24
Zheng et al	P	2022	92	106	16/76	23/83	–	–	8.1 ±3.0	8.9 ± 3.3	MWA:12.7±4.1 Surgery:12.6±5.0

MWA, microwave ablation; M, male; F, female; P, prospective study; R, retrospective study; M, month.

### Results of quality assessment

The quality and bias risks of the listed studies were assessed using Cochrane Risk of Bias 2.0 (21). Six of the seven publications were retrospective studies, one was a prospective study, and all were randomized clinical trials. Three studies also had scant or no follow-up. **Table 2** displayed the summary bias evaluations of all included studies.

**Table 2:** Summary of the methodological quality assessment of the included studies.

Study	Bias arising from the randomization process	Bias due to deviations from intended interventions	Bias due to missing outcome data	Bias in measurement of the outcome	Bias in selection of the reported result	Overall bias
Li et al (14)	Some concerns	low	low	low	low	Some concerns
Xu et al (15)	Some concerns	low	low	low	low	Some concerns
Chen et al (16)	Some concerns	low	low	low	low	Some concerns
Li et al (17)	Some concerns	low	low	low	low	Some concerns
Zu et al (18)	Some concerns	low	low	low	low	Some concerns
Wang et al (19)	Some concerns	low	low	low	low	Some concerns
Zheng et al (20)	low	low	low	low	low	low

### Intraoperative results

**Figure 2:**
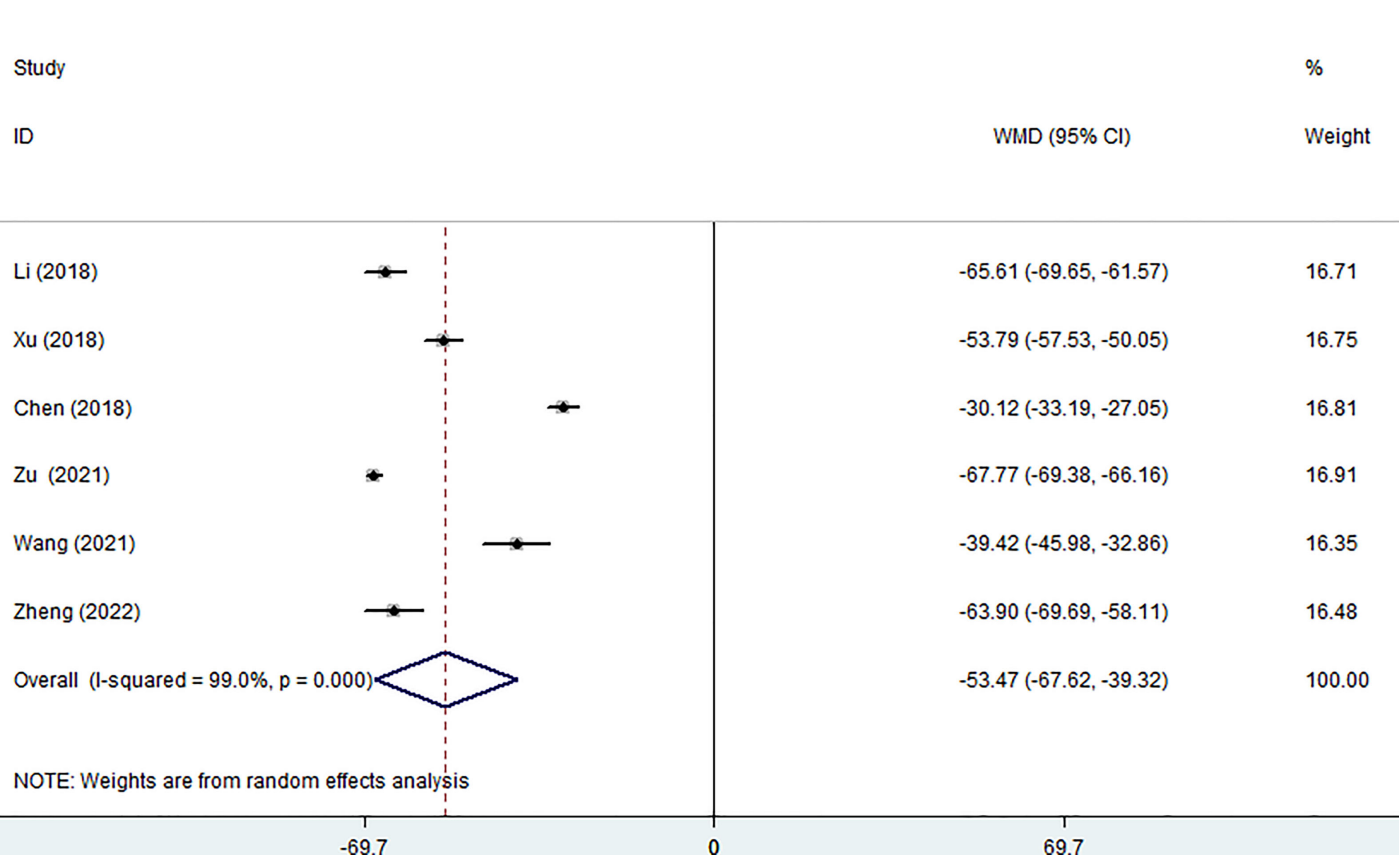
Forest plot of the operation time for comparing MWA with surgery in patient with PTMC. MWA, microwave ablation, PTMC, papillary thyroid microcarcinoma.

Six of the seven studies (including 1,256 patients) focused on operation time. In comparison to the surgery group, the MWA group took considerably less operation time (WMD =-53.47, 95% CI: -67.62 to -39.32; P<0.05; random effects model; as shown in **Figure 2**).

**Figure 3:**
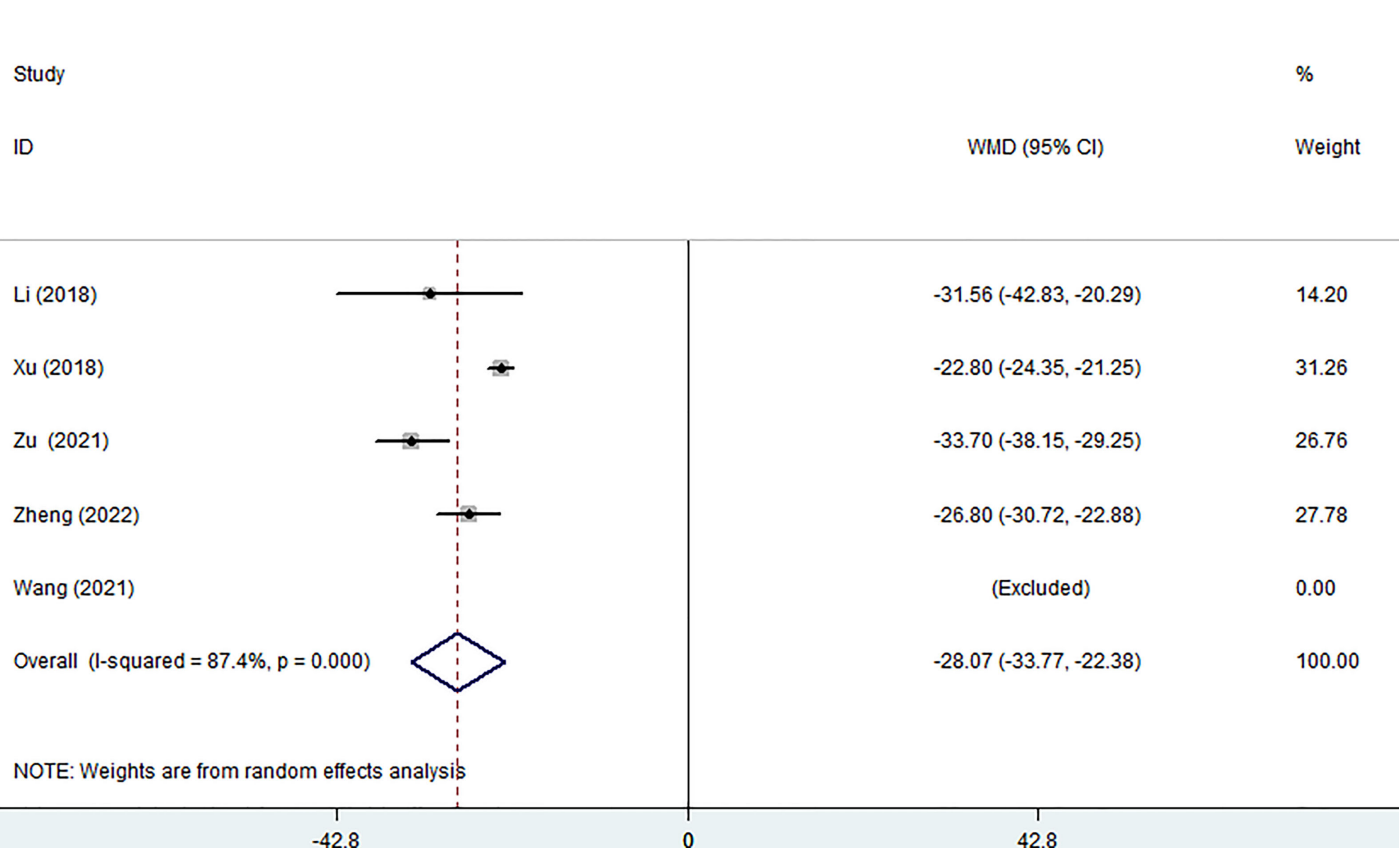
Forest plot of the blood loss for comparing MWA with surgery in patient with PTMC. MWA, microwave ablation, PTMC, papillary thyroid microcarcinoma.

Five of the seven studies examined blood loss. The pooled data showed that the MWA group lost significantly less blood than the surgical group using a random effects model. (WMD =-28.07, 95% CI: -33.77 to -22.38; P<0.05; as shown in **Figure 3**).

**Figure 4:**
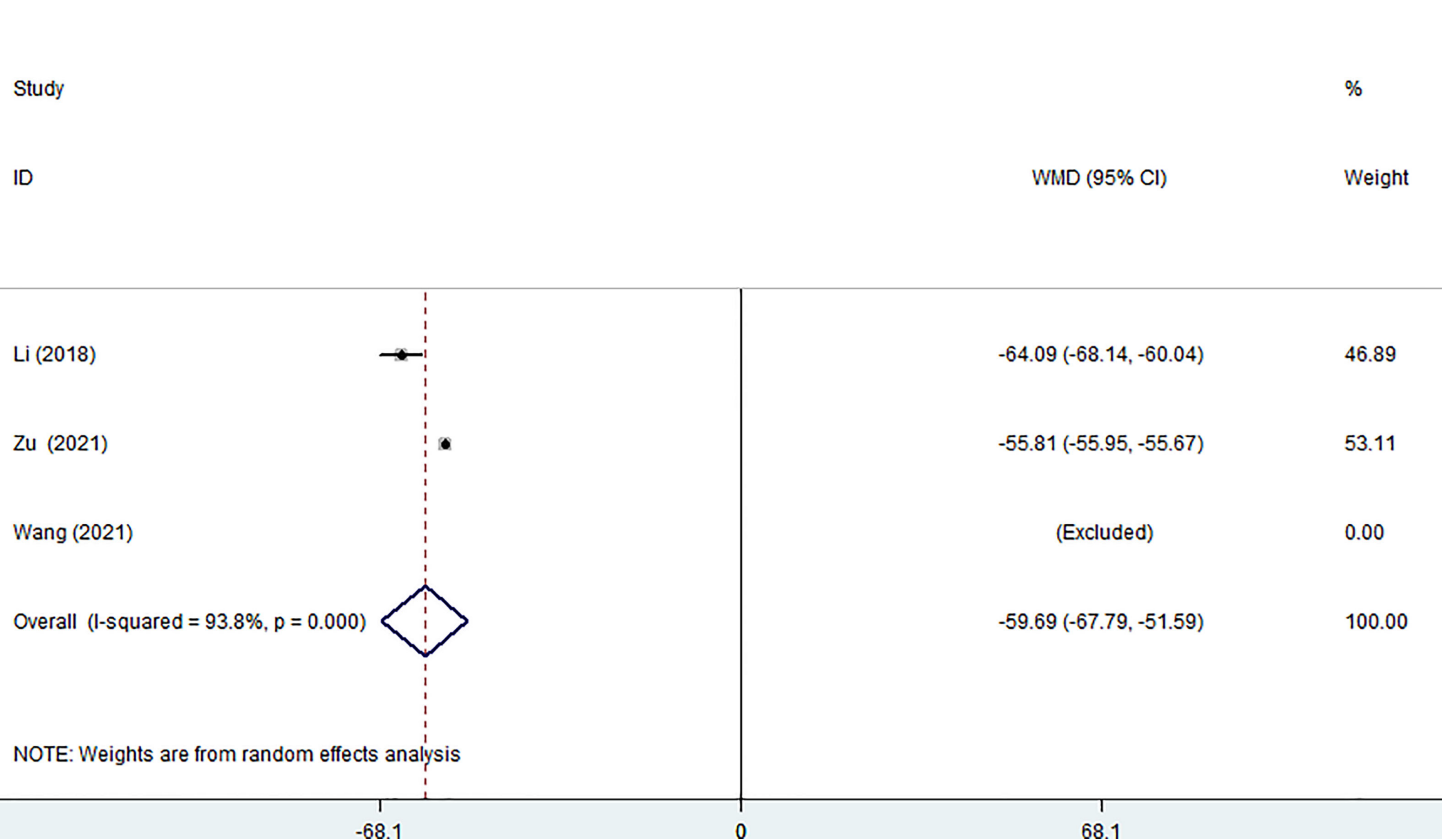
Forest plot of the size of surgical incision for comparing MWA with surgery in patient with PTMC. MWA, microwave ablation, PTMC, papillary thyroid microcarcinoma.

Three of the seven studies (including 882 patients) reported on the size of surgical incisions. The combined estimate revealed that the MWA group’s incision size was considerably smaller than that of the surgery group (WMD =-59.69, 95% CI: -67.79 to -51.59; P<0.05, random effects model; as shown in **Figure 4**).

### Post-surgical results

**Figure 5:**
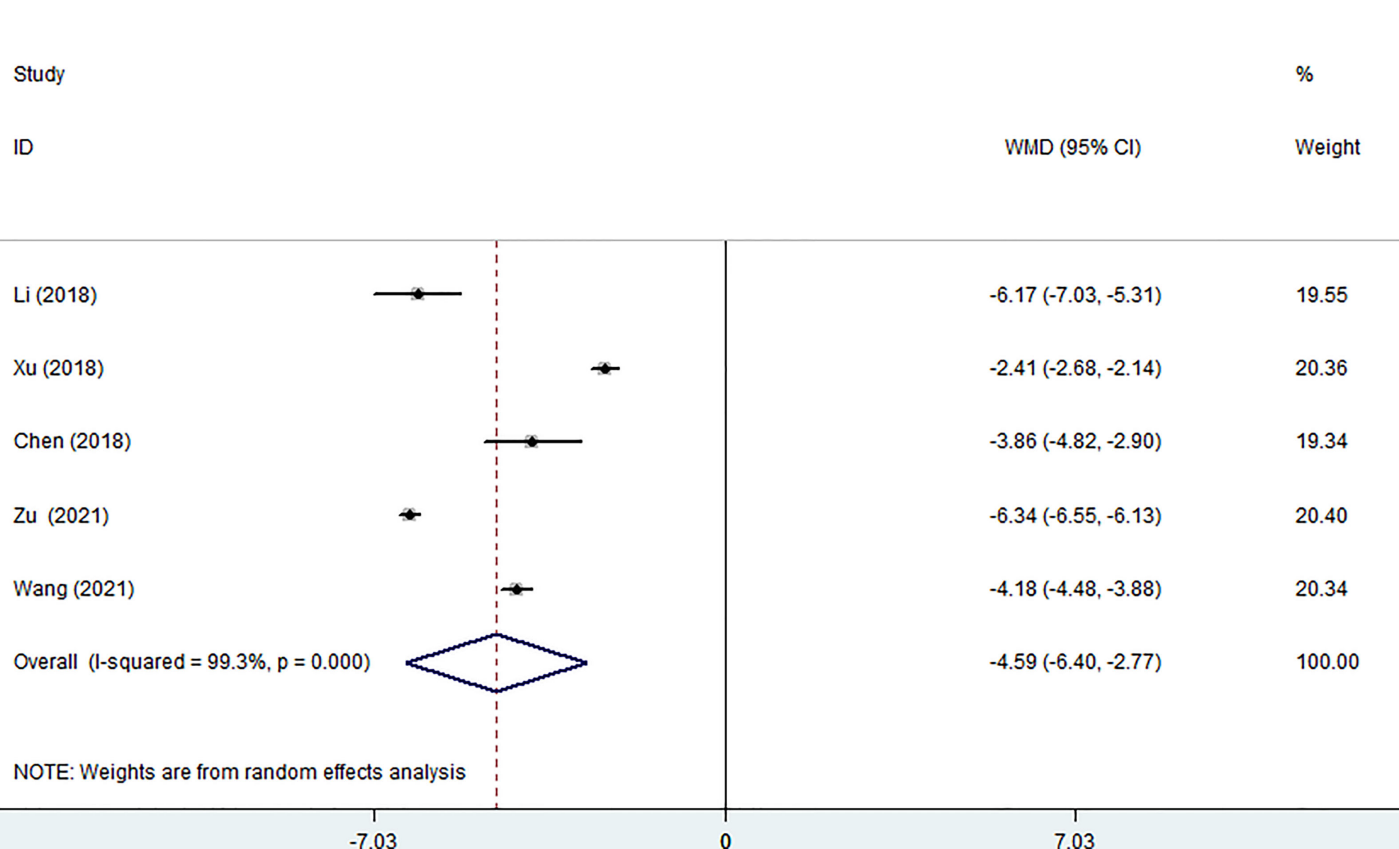
Forest plot of the hospital stay for comparing MWA with surgery in patient with PTMC. MWA, microwave ablation, PTMC, papillary thyroid microcarcinoma.

Five of the seven studies (including 1,058 patients) evaluated hospital stays. According to the combined findings, MWA significantly shortened patients’ stays in the hospital (WMD =-4.59, 95% CI: -6.40 to -2.77; P<0.05, random-effects model; as shown in **Figure 5**).

**Figure 6:**
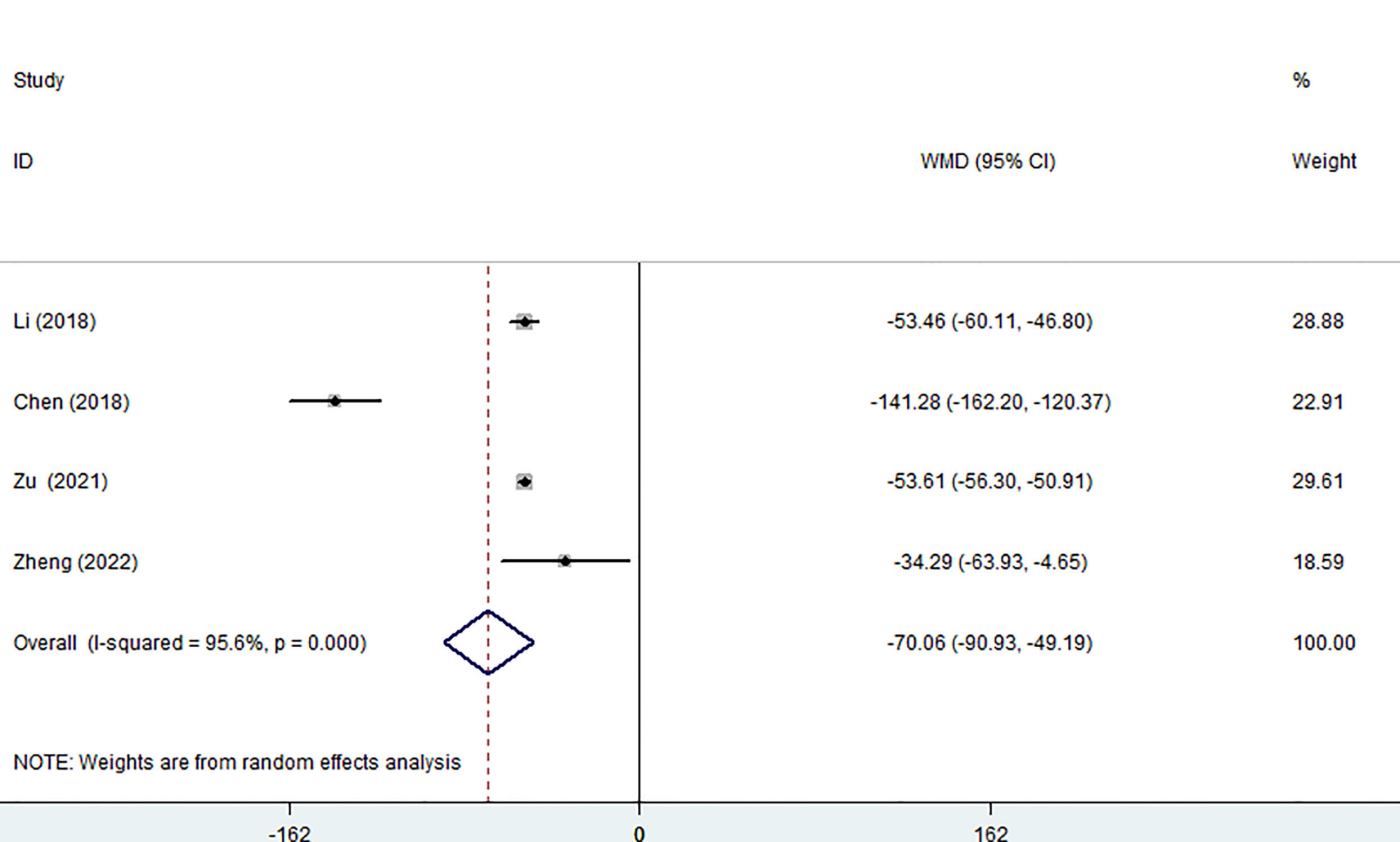
Forest plot of the hospitalization costs for comparing MWA with surgery in patient with PTMC. MWA, microwave ablation, PTMC, papillary thyroid microcarcinoma.

Four of the seven studies (including 1,023 patients) evaluated hospitalization costs. Hospitalization costs for the MWA group were considerably lower than for the surgery group, according to the pooled data (WMD= -70.06, 95% CI: -90.93 to -49.19; P<0.05, random-effects model; as shown in **Figure 6**).

### Follow-up results

**Figure 7:**
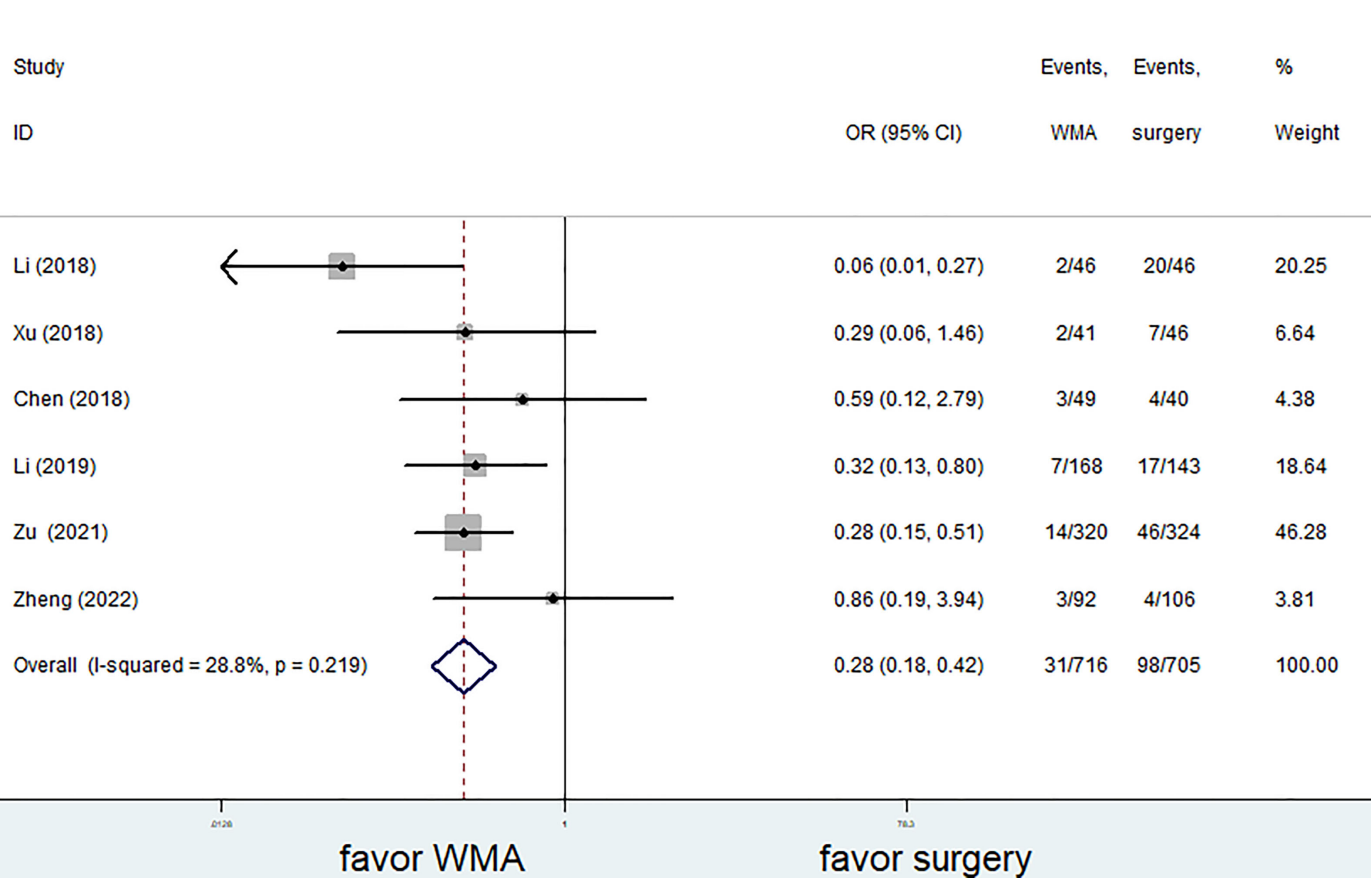
Forest plot of complications for comparing MWA with surgery in patient with PTMC. MWA, microwave ablation, PTMC, papillary thyroid microcarcinoma.

**Figure 8:**
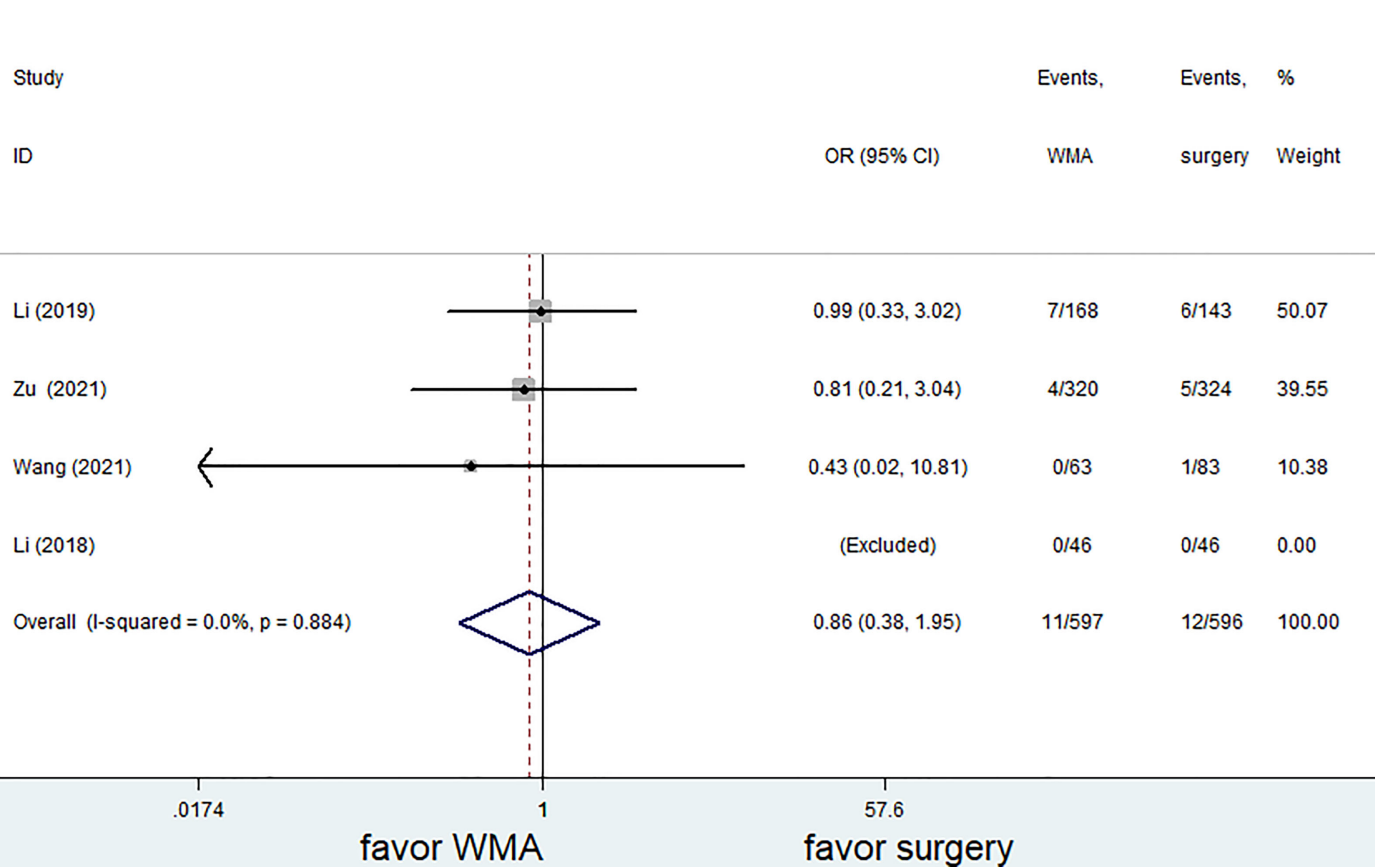
Forest plot of recurrence rates for comparing MWA with surgery in patient with PTMC. MWA, microwave ablation, PTMC, papillary thyroid microcarcinoma.

**Figure 9:**
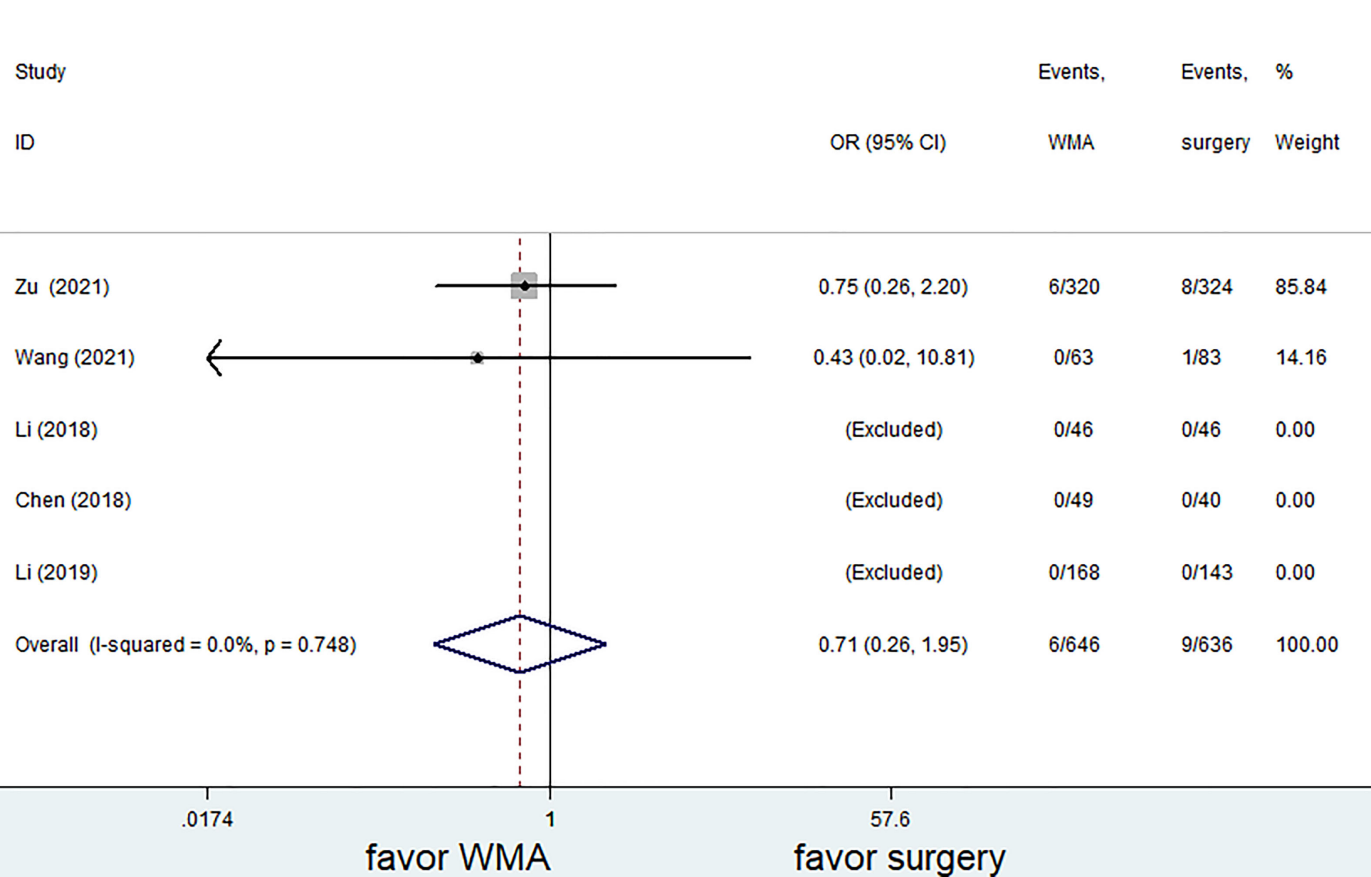
Forest plot of lymph node metastasis for comparing MWA with surgery in patient with PTMC. MWA, microwave ablation, PTMC, papillary thyroid microcarcinoma.

Six of the seven studies (1,421 patients) focused on complications. The complication rate in the MWA group was significantly lower than that in the surgical group. (OR = 0.28; 95% CI: 0.18 to 0.42; P<0.05, fixed effects model; as shown in **Figure 7**). There was no discernible difference between the MWA group and the surgery group in the four studies that assessed the recurrence rate (a total of 1,193 patients) (OR= 0.86, 95% CI: 0.38 to 1.95; P>0.05, fixed-effects model; as shown in **Figure 8**). Similar to this, a meta-analysis of the variations in lymph node metastasis between the two groups was conducted, but no appreciable difference was found. (OR =0.71, 95% CI: 0.26 to 1.95; P>0.05; as shown in **Figure 9**).

### Results of sensitivity analysis

**Figure 10:**
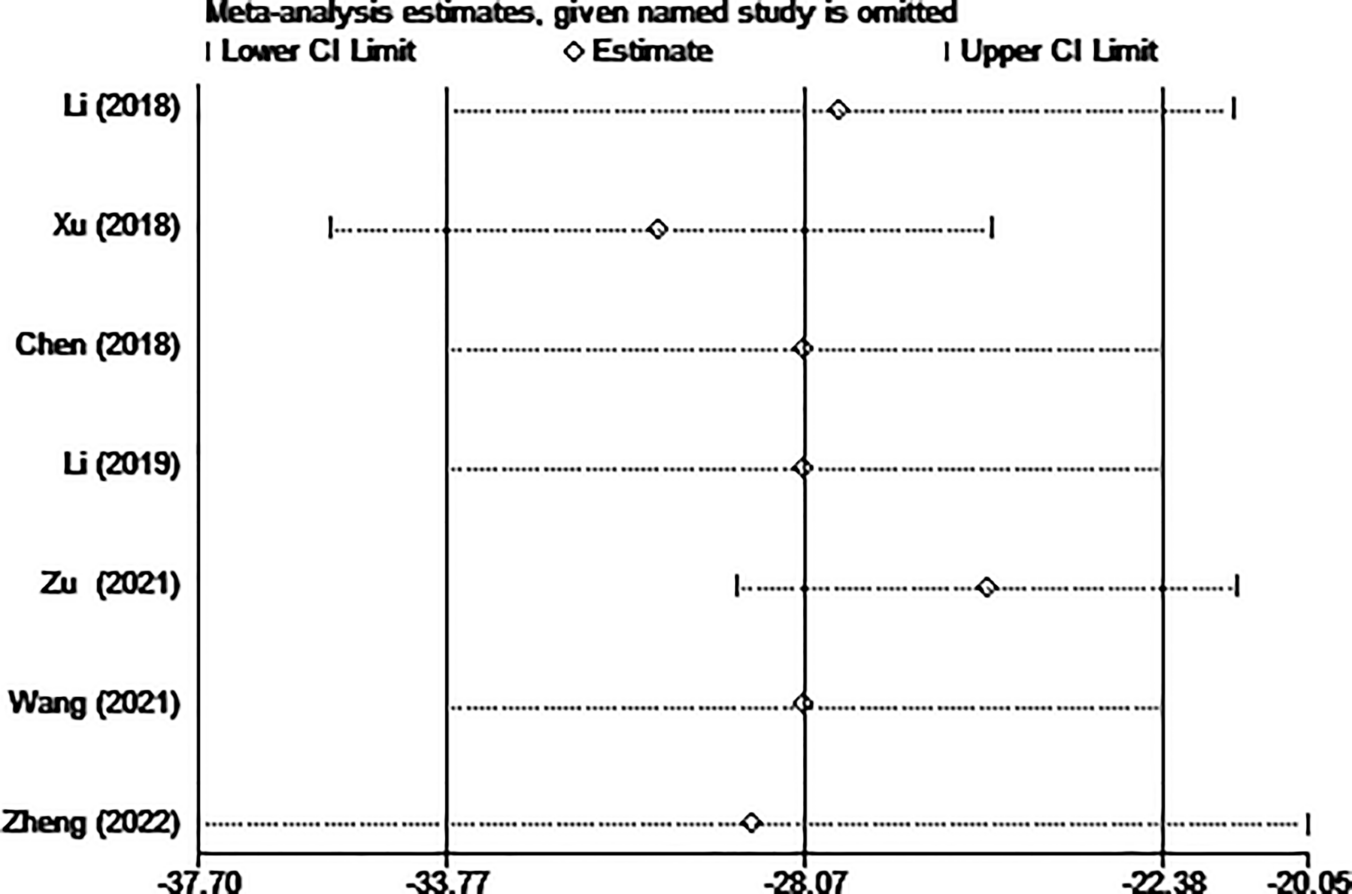
Sensitivity analysis of blood loss for comparing MWA with surgery in patient with PTMC. MWA, microwave ablation, PTMC, papillary thyroid microcarcinoma.

**Figure 11:**
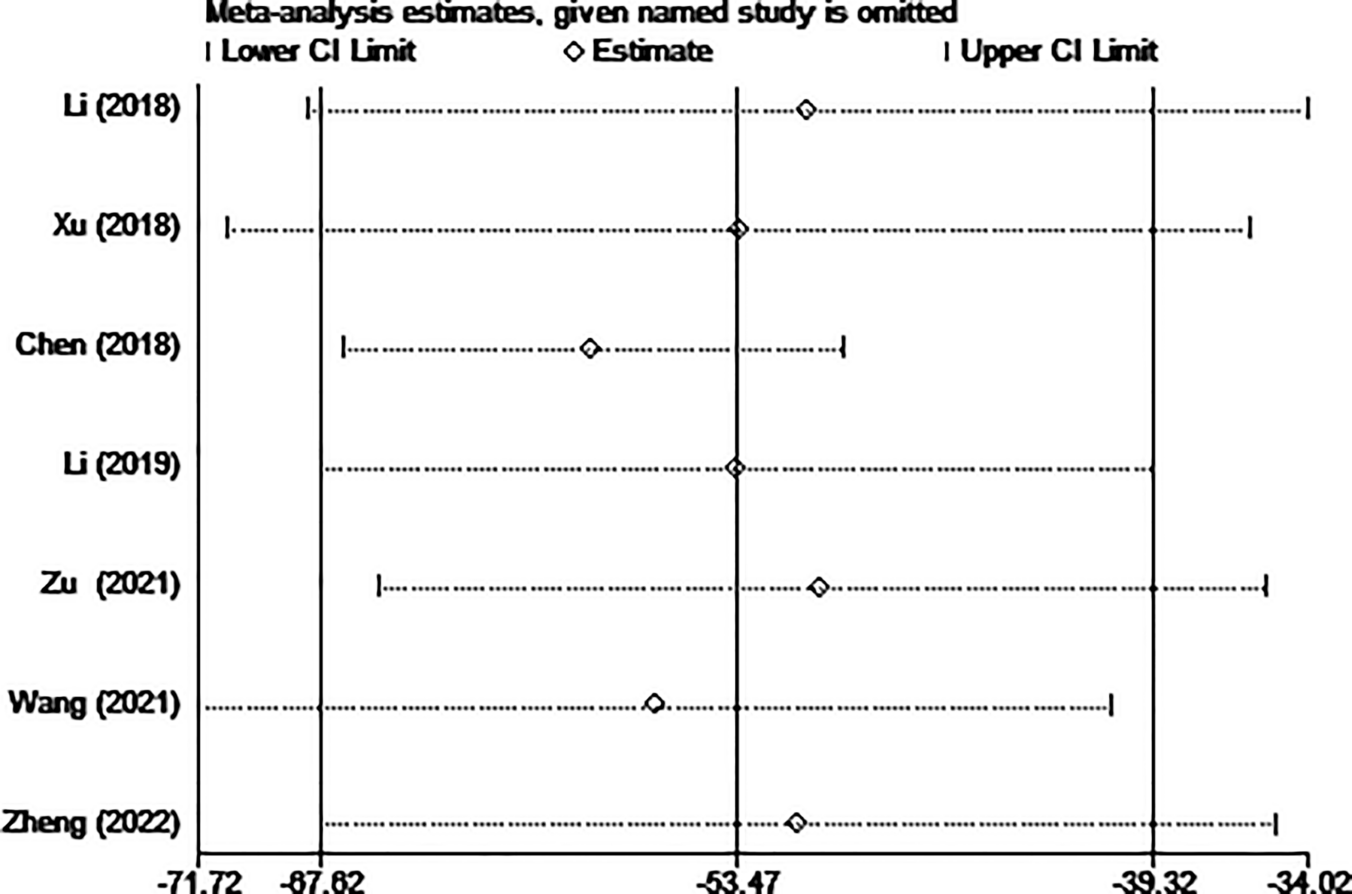
Sensitivity analysis of operation time for comparing MWA with surgery in patient with PTMC. MWA, microwave ablation, PTMC, papillary thyroid microcarcinoma.

**Figure 12:**
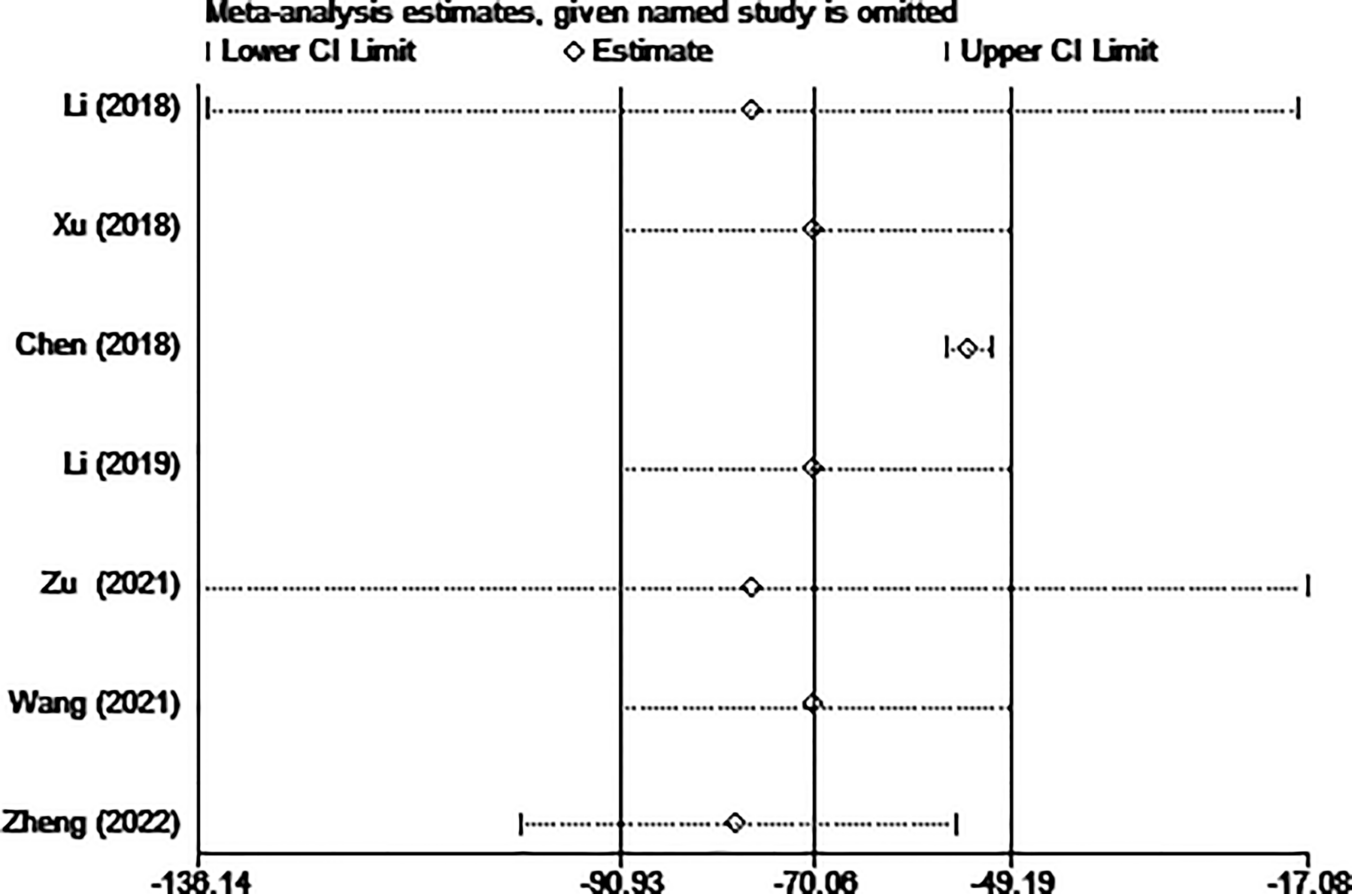
Sensitivity analysis of hospitalization costs for comparing MWA with surgery in patient with PTMC. MWA, microwave ablation, PTMC, papillary thyroid microcarcinoma.

**Figure 13:**
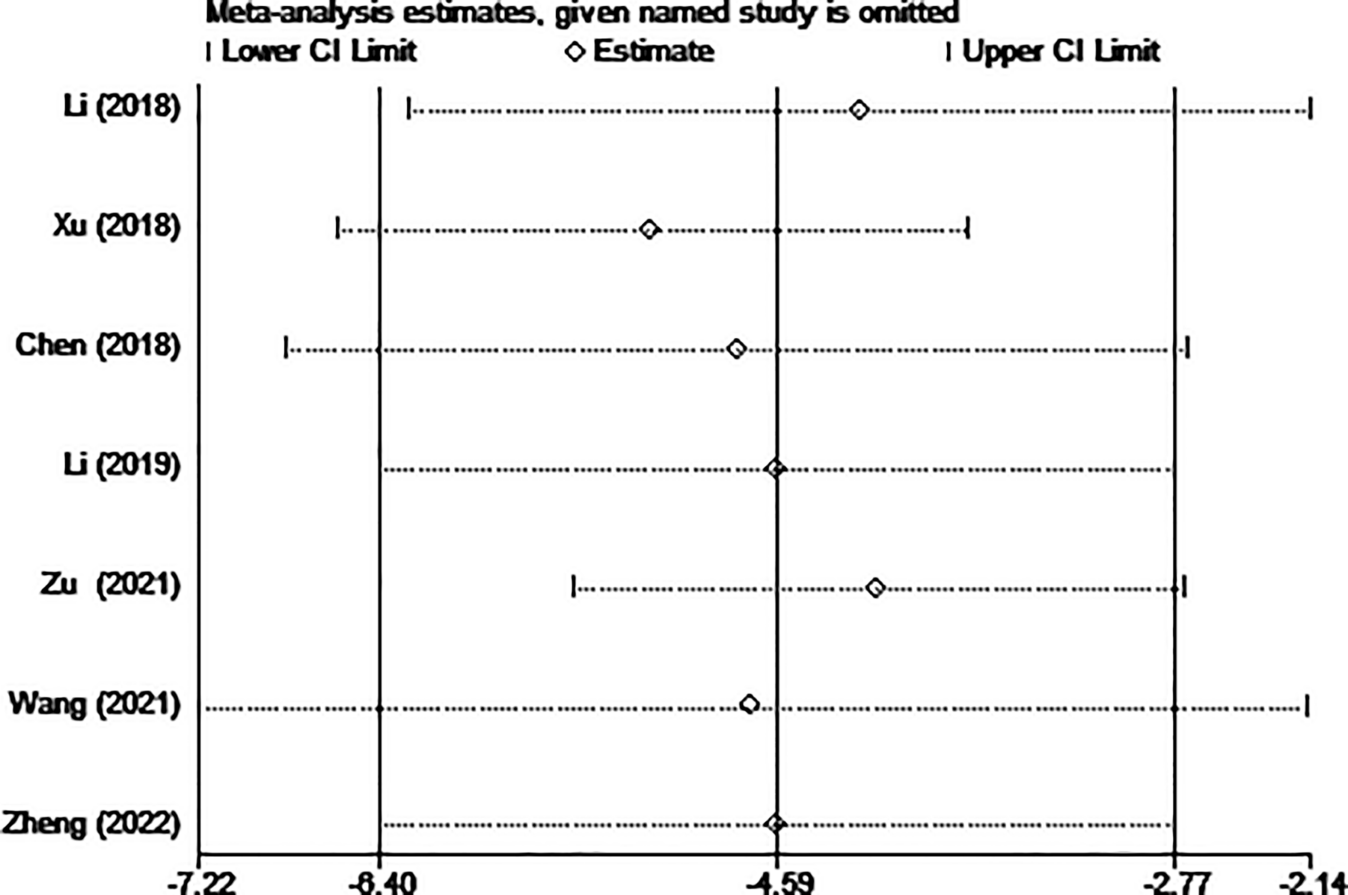
Sensitivity analysis of hospital stay for comparing MWA with surgery in patient with PTMC. MWA, microwave ablation, PTMC, papillary thyroid microcarcinoma.

The results of the sensitivity analysis are shown in **Figures 10**–**13**. The results showed that after eliminating Chen’s and Zu’s study, the recombined effect size changed greatly, so we considered Chen’s and Zu’s study studies as the possible causes of heterogeneity.

## Discussion

Surgery is known as the cornerstone of traditional treatment for patients with PTMC (22). However, it may cause a series of complications, namely hypoparathyroidism, hypothyroidism, paralysis of the recurrent laryngeal nerve, or ugly scars (23). It may have a negative impact on the patient’s quality of life, and mental health. MWA has been applied in liver, lung, kidney, and bone marrow lesions with the advancement of technology (10, 24). MWA is gaining more and more recognition in thyroid treatment (25).

In this study, we found that MWA could reduce operation time, hospital stay, hospitalization costs, incision size, blood loss, and complications without increasing the risk of recurrence and lymph node metastasis. Moreover, the latest expert consensus on thyroid thermal ablation recommends that PTMC be ablated to avoid complications after surgical treatment, such as choking on drinking water, low tone, hoarse voice, difficulty in breathing (26).

Recurrent laryngeal nerve damage following thyroid surgery has been found to occur at a rate of 0.3%-15.4% (27). The common causes of recurrent laryngeal nerve injury include tumor adhesion, nerve invasion, surgical operation, and so on. Damage to the recurrent laryngeal nerve may result in hoarseness and breathing difficulties (28). After surgery, the patient might have a deep voice and water choking if the superior laryngeal nerve has been injured. In addition, hypothyroidism and hypocalcemia following surgery are quite frequent. The incidence of hypothyroidism is reported to be as high as 75%, while the incidence of permanent hypocalcemia ranges from 0% to 3.1% (29, 30).

The duration of the procedure is also a factor in the safety equation because, in some aspects, a lengthy procedure increases the risk of bleeding, tissue damage, and anesthesia complications. Our research revealed that the operation time of the MWA group was significantly shorter than that of the surgery group (p<0.05). The primary factor is that a portion of MWA can be carried out as an outpatient surgery, and the rapid turnaround time means that general anesthesia is not required. In addition, postoperative hospital stay and perioperative costs were also compared, and the differences were statistically significant. We found that the MWA group had shorter hospital stays and lower costs after treatment, suggesting that PTMC patients recovered more quickly after surgery. In considering the medical resources and the cost of patient care, especially the high incidence of PTMC, MWA may be a better choice for PTMC patients. MWA has the above advantages for the following reasons: firstly, MWA is treated with fine needles, which means it is more discrete and straightforward to approach the aim. In addition, the energy deployment of MWA is predictable and precise. The relatively small necrotic area after MWA means faster recovery, shorter hospital stays, and ultimately lower costs (8, 31). Both results (I^2 =^ 99.3%, I^2 =^ 95.6%) demonstrated high heterogeneity, which might be brought on by an imbalance in how different regions are developing economically, the difference in medical technology level, and the experience of different surgeons.

Since MWA is treated with ablation needles, the incision is naturally smaller than surgery. Besides, our study also found that the intraoperative blood loss of MWA was less than that of surgery, which indicated that MWA was less traumatic to the human body and more conducive to prevent the occurrence of postoperative neck hematoma, so as to avoid the risk of asphyxia caused by hematoma compression of the trachea. Additionally, because to the fibrosis brought on by scar tissue and the distortion of postoperative normal tissue, repeated surgery for tumor recurrence after thyroid surgery is usually difficult (17, 32). Therefore, postoperative tumor recurrence is also an important follow-up index. Some scientists advocated that compared with surgery, MWA may lead to incomplete treatment and therefore a higher rate of recurrence, enduring or even remote metastases during extended follow-up (33). However, our findings revealed that there was no statistically significant distinction between individuals who underwent MWA and surgery in terms of tumor recurrence or lymph node metastasis. Consequently, a randomized controlled trial (RCT) with a large sample size and lengthy follow-up period is urgently needed to confirm whether there are differences between MWA and surgery in recurrence and lymph node metastasis after PTMC. Although there are many advantages about MWA, there are also disadvantages. The disadvantages of MWA are mainly as follows: Firstly, it is impossible to make pathological judgment, and even if there is “puncture”, the diagnosis is not comprehensive and reliable. The understanding of benign, malignant and metastasis of thyroid tumors is insufficient. Besides, it cannot provide three-dimensional observation and judgment and all-round understanding and control of the tumor and its surrounding tissues during operation. Finally, it is only applicable to low-risk PTMC, not to other types of thyroid cancer.

Our study has the following limitations, first of all, all the included studies focused on China. The results of our meta-analysis may not be comparable to that in other areas where medical technology and levels may differ because of our study’s lack of extensibility. The results would be more consistent if high-quality research were carried out in the United States, Europe, or any other nations and included in this meta-analysis. Second, the number of the included studies were relatively limited, with most studies being retrospective cohort studies. If RCTs were included, the meta-overall analysis’s result would be more trustworthy. Third, there is considerable range in operation duration, postoperative hospitalization, and perioperative expenditures, which may be a result of regional economic conditions, variations in medical training, and patient age differences. Subgroup analysis couldn’t be done since there weren’t enough studies included. The source of heterogeneity could be found if more comparable studies were conducted and included in the meta-analysis. Fourth, MWA for PTMC is a relatively new technique with a limited mean follow-up time. Studies with extended follow-up are required to determine whether the findings are enduring or repeatable. Preferably more than five years of follow-up.

In conclusion, our meta-analysis showed that MWA had a considerable reduction in the rate of complications, operative time, postoperative hospital stay, and hospitalization costs compared with surgery. It also showed a recurrence rate and risk of lymph node metastasis similar to surgery. MWA may therefore be an efficient, secure, and cost-effective treatment for low-risk PTMC patients.

## Data availability statement

The raw data supporting the conclusions of this article will be made available by the authors, without undue reservation.

## Author contributions

JF designed the study. JF and YJ pooled the data. JF and YF analyzed the data. JF wrote and reviewed the article. All authors contributed to the article and approved the submitted version.
